# SHAP-based interpretable machine learning for Parkinson's disease severity prediction: integrated analysis of clinical and environmental features

**DOI:** 10.3389/fneur.2025.1678463

**Published:** 2025-09-29

**Authors:** Yuting Jin, Xiang Li, Xinsheng Han, Yang Qiu, Hongyang Zhang, Jianke Xu, Miao Han

**Affiliations:** ^1^Department of Neurology, Kaifeng Central Hospital, Kaifeng, Henan, China; ^2^College of Information Engineering, Yellow River Conservancy Technical University, Kaifeng, Henan, China

**Keywords:** Parkinson's disease, machine learning, disease severity, environmental factors, SHAP, interpretable artificial intelligence

## Abstract

**Introduction:**

Parkinson's Disease (PD) represents the second most prevalent neurodegenerative disorder globally, with traditional assessment methods suffering from limitations including substantial inter-rater variability and inability to capture multifactorial complexity underlying disease progression.

**Methods:**

Based on data from 500 Parkinson's disease patients, we integrated 7 standardized clinical phenotypes (excluding UPDRS to prevent data leakage) and 8 environmental exposure factors, compared 10 machine learning algorithms using 5-fold cross-validation, and applied SHAP interpretability analysis for transparent feature importance assessment.

**Results:**

XGBoost with SMOTE sampling achieved clinically meaningful discriminative performance (AUC = 0.781, precision = 0.548, recall = 0.750) appropriate for screening applications. SHAP analysis revealed non-motor symptoms as the primary predictor (SHAP value = 2.76), followed by serum dopamine concentration (2.39) and age (2.16), while environmental factors demonstrated modest but statistically significant contributions.

**Discussion:**

This proof-of-concept study developed an interpretable framework with methodological safeguards against data leakage, demonstrating promising screening potential with realistic performance expectations. However, the cross-sectional, single-center design limits generalizability, requiring external validation and longitudinal studies before clinical deployment.

## 1 Introduction

Parkinson's Disease (PD) represents the second most prevalent neurodegenerative disorder globally, affecting over 10 million individuals worldwide (1), with increasing prevalence driven by population aging (2). This exponential growth trajectory, driven primarily by population aging, positions PD severity assessment as a critical bottleneck in modern healthcare delivery and resource optimization (2). The clinical imperative for precise severity stratification extends beyond individual patient care to encompass broader healthcare system planning, treatment resource allocation, and long-term care projections. Traditional assessment paradigms, predominantly anchored on the Unified Parkinson's Disease Rating Scale (UPDRS) and Hoehn-Yahr staging systems, while clinically established, suffer from inherent limitations including substantial inter-rater variability, subjective interpretation bias, and inability to capture the multifactorial complexity underlying disease progression (3, 4). These methodological constraints become particularly pronounced when attempting to integrate the growing body of evidence suggesting that environmental factors—ranging from meteorological conditions and air quality to ultraviolet exposure—may significantly modulate dopaminergic neuronal function through oxidative stress pathways and neuroinflammatory cascades (5, 6). The interplay between intrinsic clinical phenotypes and extrinsic environmental exposures represents a critical yet underexplored dimension in PD severity prediction, demanding innovative methodological approaches that can simultaneously capture disease complexity while maintaining clinical interpretability and methodological rigor.

Current research landscapes reveal three fundamental gaps that collectively impede the development of comprehensive PD severity assessment tools. First, existing machine learning applications in PD research predominantly adopt single-modality approaches, focusing exclusively on motor symptoms (7) without systematically integrating the multidimensional clinical-environmental feature space that more accurately reflects disease etiology and progression mechanisms. This reductionist approach fails to capture the complex interactions between genetic predisposition, clinical manifestations, and environmental modulators that collectively determine disease severity trajectories. Second, the pervasive “black box” problem inherent in contemporary machine learning applications severely limits clinical adoption, as healthcare practitioners require transparent, interpretable decision-making frameworks to establish trust, validate clinical intuition, and make informed therapeutic decisions. The absence of explainable AI methodologies in PD research creates a critical translation barrier between algorithmic prediction capability and clinical utility. Third, despite mounting epidemiological evidence suggesting environmental factors significantly influence PD onset and progression (2), quantitative assessment of these factors' contributions to severity prediction remains largely unexplored, limiting the development of evidence-based environmental intervention strategies and personalized risk management approaches.

Therefore, our research addresses these fundamental limitations through: (1) developing a machine learning framework that integrates clinical quantitative phenotypes with environmental meteorological factors for PD severity prediction; (2) implementing advanced SHAP (SHapley Additive exPlanations) interpretability analysis to provide transparent, feature-specific decision rationale that enhances clinical trust and enables individualized patient assessment; (3) quantitatively characterizing the relative contributions of environmental factors to disease severity prediction, providing empirical foundation for environmental intervention strategies and precision medicine applications in PD management.

The remainder of this manuscript is organized as follows: Section 2 presents our comprehensive methodology including ethical frameworks, data collection protocols, target variable reconstruction procedures to address methodological concerns, machine learning algorithms, and interpretability analysis techniques; Section 3 details our systematic results including data leakage risk assessment, corrected model performance analysis, comprehensive sampling strategy evaluation, and SHAP-based interpretability findings; Section 4 discusses the clinical implications of our methodological innovations, pathophysiological insights from feature importance analysis, study limitations, and future research directions; and Section 5 summarizes our key contributions and their potential impact on PD clinical practice and research paradigms.

## 2 Methods

This study represents a proof-of-concept development and internal validation of an interpretable machine learning framework. The single-center design was chosen for initial methodology development and feasibility assessment. While internal cross-validation demonstrates model stability within our population, external validation across independent datasets is required to assess true generalizability. The performance metrics reported should be interpreted as preliminary estimates requiring confirmation in diverse clinical settings.

### 2.1 Study design and ethical approval

This study adopted a cross-sectional observational study design and was approved by the Medical Ethics Committee of Kaifeng Central Hospital (Ethics approval number: 2021ks-kt021), strictly following the Declaration of Helsinki and relevant regulatory requirements. All participating patients signed informed consent forms, and the data collection process implemented de-identification to ensure patient privacy and security.

**Figure 1:**
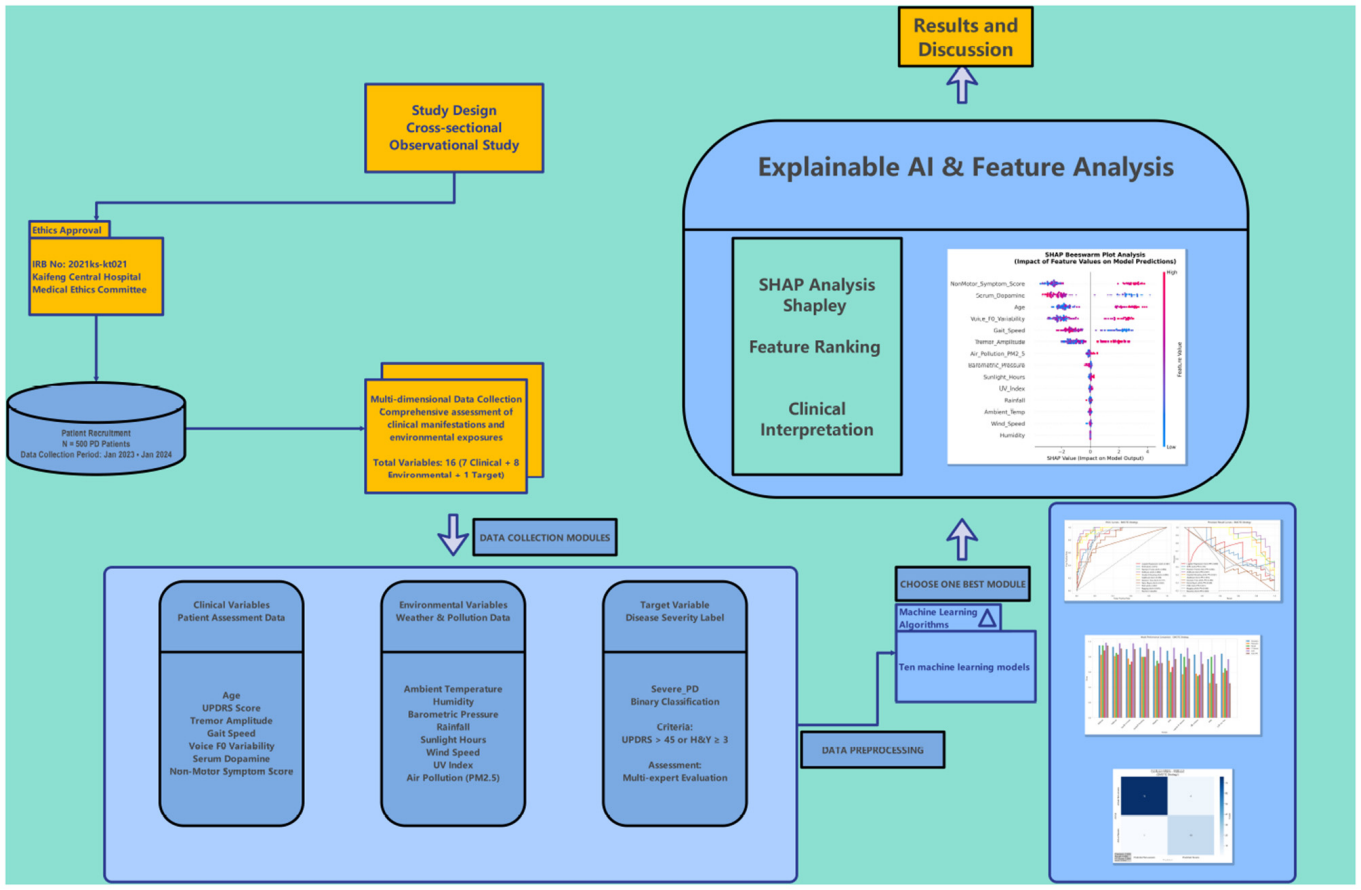
Study design framework. This flowchart shows the complete research framework from study design, ethical approval, patient recruitment, multi-dimensional data collection, machine learning modeling to interpretability analysis. The **left side** shows the three core modules of data collection (clinical variables, environmental variables, target variables), and the **right side** shows the machine learning analysis process and SHAP interpretability analysis results.

As shown in Figure 1, the overall framework of this study includes four core modules: study design module, data collection module, machine learning algorithm module, and interpretable AI analysis module.

The study began with ethical approval, screening 500 Parkinson's disease patients through strict inclusion criteria, adopting a cross-sectional observational study design for data collection. The data collection module covered comprehensive assessment of 16 variables, including 7 clinical characterization variables, 8 environmental exposure variables, and 1 disease severity target variable. In the machine learning algorithm module, we selected 10 classical algorithms for modeling comparison, selecting the optimal model through data preprocessing, 5-fold cross-validation, and multi-indicator evaluation. Finally, SHAP analysis was used for interpretable AI feature analysis to form clinical interpretation results.

### 2.2 Study subjects and inclusion criteria

Study subjects were Parkinson's disease patients treated at the Neurology Department of Kaifeng Central Hospital from January 2023 to January 2024. Based on prior statistical power analysis, setting effect size at 0.5, test level α = 0.05, and test power 1−β = 0.80, the minimum sample size was calculated as 394 cases. Considering a 20% dropout rate, the final sample size was determined as 500 cases.

**Inclusion criteria:**

1. Primary Parkinson's disease patients meeting the UK Parkinson's Disease Society clinical diagnostic criteria, with at least two major motor symptoms (resting tremor, muscle rigidity, bradykinesia, postural gait abnormalities) (8);
2. Age 18–85 years, disease duration ≥ 6 months to ensure stable disease characteristics (9);
3. Basically intact cognitive function (MMSE score ≥ 24), able to cooperate in completing scale assessments (10);
4. Relatively stable medication regimen within the past 3 months to avoid the impact of acute medication adjustments on assessment results (3);
5. Clear and stable residential address for accurate matching of environmental exposure data.

**Exclusion criteria:**

1. Secondary parkinsonism or Parkinson-plus syndromes (9);
2. Severe cognitive dysfunction (MMSE < 24) or psychiatric disorders affecting assessment (10);
3. Missing important clinical data exceeding 20% (11, 12);
4. Concurrent severe heart, liver, kidney dysfunction or malignant tumors;
5. Recent deep brain stimulation or other surgical treatments (9).

### 2.3 Data collection system

#### 2.3.1 Clinical variable collection (7 core variables)

Clinical data collection strictly followed standardized operating procedures, completed collaboratively by attending neurologists and professional technicians with more than 5 years of experience. All assessors received 3 days of standardized training with inter-rater reliability coefficient ≥0.85.

Specific collection variables include:

1. **Age**: Patient's actual age in years, extracted through medical record systems and verified with ID information.
2. **UPDRS score**: Total score of Unified Parkinson's Disease Assessment Scale Parts I-IV, scoring range 0–199. Independently assessed by 2 attending neurologists, taking the average to reduce assessment bias.
3. **Tremor amplitude**: Resting tremor amplitude assessment, using UPDRS Part III manual scoring combined with triaxial accelerometer objective measurement, scoring range 0-4 levels.
4. **Gait speed**: 10-meter straight-line walking speed measurement in m/s. Measured by rehabilitation therapists using standardized gait analyzers in barrier-free corridors.
5. **Voice F0 variability**: Fundamental frequency variation coefficient in %. Collected patients' sustained “ah” sound for 5 seconds, analyzed using Praat 6.3 software.
6. **Serum dopamine**: Serum dopamine concentration in ng/mL. Morning fasting blood collection of 5mL, detected using enzyme-linked immunosorbent assay (ELISA).
7. **Non-motor symptom score**: Using Chinese version Non-Motor Symptoms Scale (NMSS), total score range 0–360.

#### 2.3.2 Environmental variable collection (8 exposure variables)

Environmental data collection adopted a multi-source data fusion strategy to ensure data accuracy and completeness. Major data sources include China Meteorological Administration National Meteorological Information Center (NMIC), National Climate Center (NCC), NOAA Global Surface Summary of Day (GSOD), National Ministry of Ecology and Environment Air Quality Monitoring Network, and NASA satellite remote sensing data.

**Spatiotemporal matching algorithm:** Considering the lag effects of environmental factors on the body, this study established a precise spatiotemporal matching algorithm. The spatial matching used distance-weighted interpolation method as shown in Equation 1 (13, 14):


(1) $$\begin{array}{l} E_{i}=\frac{\sum_{j=1}^{n}w_{j}\cdot E_{j}}{\sum_{j=1}^{n}w_{j}} \end{array}$$

where *E*_*i*_ is the environmental exposure estimate for patient *i*, *E*_*j*_ is the environmental measurement at monitoring station *j*, and *w*_*j*_ is the distance weight calculated as:


(2) $$\begin{array}{l} w_{j}=\frac{1}{d_{ij}^{2}} \end{array}$$

where *d*_*ij*_ is the distance between patient *i*

Specific environmental variables include (5, 6):

(1) **Ambient temperature**: Degrees Celsius (°C), average daily temperature 7 days before patient visit.
(2) **Humidity**: Percentage (%), average relative humidity 7 days before visit.
(3) **Barometric pressure**: Hectopascals (hPa), average atmospheric pressure on visit day.
(4) **Rainfall**: Millimeters (mm), cumulative precipitation 7 days before visit.
(5) **Sunlight hours**: Hours (h), average sunshine duration 7 days before visit.
(6) **Wind speed**: Meters per second (m/s), average wind speed 3 days before visit.
(7) **UV index**: Dimensionless index, maximum UV index 3 days before visit.
(8) **PM2.5 concentration**: Micrograms per cubic meter (μg/m^3^), average PM2.5 concentration 7 days before visit.

#### 2.3.3 Environmental exposure assessment limitations

The selection of temporal lag windows (7 days for most meteorological variables, 3 days for wind speed and UV index) represents reasonable approximations based on available evidence of environmental health impacts on neurological conditions (5, 6), but the optimal exposure assessment windows remain uncertain. Sensitivity analyses across multiple lag periods were not performed in this dataset, and current exposure estimates should be interpreted cautiously. The biological mechanisms underlying environment-PD interactions are incompletely understood, and our temporal window selections reflect clinical approximations rather than established biological constants (15, 16).

#### 2.3.4 Target variable definition

Severe Parkinson's Disease (Severe_PD) is defined as a binary classification label (0 = non-severe, 1 = severe), based on comprehensive multi-dimensional clinical quantitative criteria. The determination criteria adopt a hierarchical assessment system to ensure classification accuracy and clinical significance.

**Primary determination criteria (meeting any one):**

1. UPDRS total score >45, indicating moderate to severe motor dysfunction;
2. Hoehn-Yahr stage ≥3, suggesting significant postural instability;
3. Significantly limited daily living ability, ADL score <70.

**Auxiliary determination criteria (meeting 2 or more):**

1. Presence of obvious motor complications including on-off phenomena, dyskinesia;
2. L-DOPA daily dose >600mg or advanced therapy initiated (DBS, pump therapy);
3. Severe non-motor symptoms, NMSS total score >70;
4. Cognitive decline, MoCA score <22.

### 2.4 Machine learning modeling

#### 2.4.1 Algorithm selection and theoretical foundation

This study selected 10 classical machine learning algorithms with different learning mechanisms to ensure comprehensiveness and scientific rigor of model comparison:

1. **Logistic regression**: Probability classification model based on linear regression, with decision function shown in Equation 3 (17):
  (3) $$\begin{array}{l} P\left(y=1|x\right)=\frac{1}{1+e^{-\left(\beta_{0}+\sum_{i=1}^{p}\beta_{i}x_{i}\right)}} \end{array}$$
2. **Support vector machine (SVM)**: Based on structural risk minimization principle, using kernel functions for nonlinear classification as in Equation 4 (18):
  (4) $$\begin{array}{l} f\left(x\right)=\text{sign}\left(\overset{n}{\underset{i=1}{\sum }}\alpha_{i}y_{i}K\left(x_{i},x\right)+b\right) \end{array}$$
3. **K-nearest neighbors (KNN)**: Instance-based learning method with prediction formula in Equation 5 (19):
  (5) $$\begin{array}{l} \hat{y}=\arg\underset{c}{\max}\underset{x_{i}\in N_{k}\left(x\right)}{\sum }I\left(y_{i}=c\right) \end{array}$$
4. **Decision tree**: Tree-form classifier based on information gain or Gini impurity (20).
5. **Random forest**: Ensemble learning method combining multiple decision trees as shown in Equation 6 (21):
  (6) $$\begin{array}{l} \hat{y}=\frac{1}{B}\overset{B}{\underset{b=1}{\sum }}T_{b}\left(x\right) \end{array}$$
6. **Gradient boosting**: Sequential ensemble method, progressively fitting residuals (22).
7. **AdaBoost**: Adaptive boosting algorithm, dynamically adjusting sample weights (23).
8. **Bagging**: Parallel ensemble learning method (24).
9. **Naive Bayes**: Probabilistic classifier based on Bayes theorem (25).
10. **XGBoost**: Extreme gradient boosting algorithm, optimized gradient boosting framework (26).

#### 2.4.2 Data preprocessing

**Outlier detection:** Using boxplot and Z-score methods to identify outliers. The Z-score is calculated using Equation 7:


(7) $$\begin{array}{l} Z=\frac{x-\mu }{\sigma } \end{array}$$

Data points with |*Z*| > 3 were marked as outliers (27).

**Missing value handling:** Different strategies based on missing mechanisms (28):

- Missing Completely at Random (MCAR): Mean imputation
- Missing at Random (MAR): Multiple Imputation by Chained Equations (MICE)
- Missing Not at Random (MNAR): Professional knowledge-based imputation

**Feature standardization:** Continuous variables using Z-score standardization as shown in Equation 8 (29):


(8) $$\begin{array}{l} x_{\text{norm}}=\frac{x-\mu }{\sigma } \end{array}$$

#### 2.4.3 Model training and validation

Stratified 5-fold cross-validation was adopted to ensure stability and reliability of model evaluation. The dataset was stratified sampled according to Severe_PD labels, ensuring consistent class distribution in each fold with the overall distribution.

### 2.5 Model interpretability analysis

#### 2.5.1 SHAP theoretical foundation

SHapley Additive exPlanations (SHAP) method (30, 31) was adopted for model interpretability analysis. SHAP is based on the Shapley value concept from game theory, representing model output as the sum of feature contributions as shown in Equation 9:


(9) $$\begin{array}{l} f\left(x\right)=\phi_{0}+\overset{M}{\underset{i=1}{\sum }}\phi_{i} \end{array}$$

where ϕ_0_ is the baseline value (average prediction of all training samples), ϕ_*i*_ is the SHAP value of the *i*-th feature, representing the marginal contribution of that feature to the prediction result.

The Shapley value calculation formula is given by Equation 10:


(10) $$\begin{array}{l} \phi_{i}=\underset{S\subseteq N\backslash \left\{i\right\}}{\sum }\frac{|S|!\left(M-|S|-1\right)!}{M!}\left[f_{x}\left(S\cup \left\{i\right\}\right)-f_{x}\left(S\right)\right] \end{array}$$

where *S* is a feature subset, *N* is the set of all features, and *M* is the total number of features.

#### 2.5.2 Interpretability analysis content

**Global feature importance:** Calculate the average absolute SHAP values across all samples for each feature, reflecting the importance of features in the overall model.

**Local prediction explanation:** Explain prediction results for individual samples, identifying features with the greatest impact on that sample's prediction.

**Feature interaction effects:** Analyze the impact of interactions between features on model predictions.

### 2.6 Model evaluation metrics

Multiple evaluation metrics were adopted to comprehensively evaluate model performance:


(11) $$\begin{array}{l} \text{Accuracy}\;\left(32\right)=\frac{TP+TN}{TP+TN+FP+FN} \end{array}$$

(32)


(12) $$\begin{array}{l} \text{Precision}\;\left(32\right)=\frac{TP}{TP+FP} \end{array}$$

(32)


(13) $$\begin{array}{l} \text{Recall}\;\left(32\right)=\frac{TP}{TP+FN} \end{array}$$

(32)


(14) $$\begin{array}{l} \text{F1-Score}\;\left(33\right)=2\times \frac{\text{Precision}\times \text{Recall}}{\text{Precision}+\text{Recall}} \end{array}$$

(33)

Additionally, the AUC (Area Under the ROC Curve) was calculated to reflect overall classification performance, where TP (True Positive), TN (True Negative), FP (False Positive), and FN (False Negative) (34, 35).

### 2.7 Statistical analysis

Python 3.11 was used for data analysis, with main software packages including:

- Data processing: Pandas 2.0, NumPy 1.24
- Machine learning: Scikit-learn 1.3, XGBoost 1.7
- Interpretability analysis: SHAP 0.42
- Data visualization: Matplotlib 3.7, Seaborn 0.12

Statistical description: Continuous variables expressed as mean ± standard deviation ($\bar{x}\pm s$), categorical variables as frequency and percentage (n, %).

Statistical inference: Independent samples t-test for comparing continuous variables between groups, chi-square test for categorical variables. Significance level set at *P* < 0.05, all tests were two-sided.

## 3 Results

### 3.1 Basic characteristics of study subjects

This study successfully enrolled 500 Parkinson's disease patients meeting the inclusion criteria. The demographic characteristics showed 289 male patients (57.8%) and 211 female patients (42.2%), with a male-to-female ratio of approximately 1.37:1, consistent with the gender distribution of Parkinson's disease. The average age was 67.4 ± 11.2 years (range: 18–85 years). Disease duration analysis showed an average of 8.6 ± 5.4 years, with early-stage patients (<5 years) accounting for 38.2%, intermediate-stage (5–10 years) for 41.4%, and late-stage (>10 years) for 20.4%.

Data quality assessment showed overall completeness of 97.8%, with inter-rater reliability coefficient of 0.84. Missing data were primarily in voice F0 variability (2.4%) and serum dopamine (1.8%), handled using multiple imputation methods.

### 3.2 Data leakage risk assessment and methodological correction

**Figure 2:**
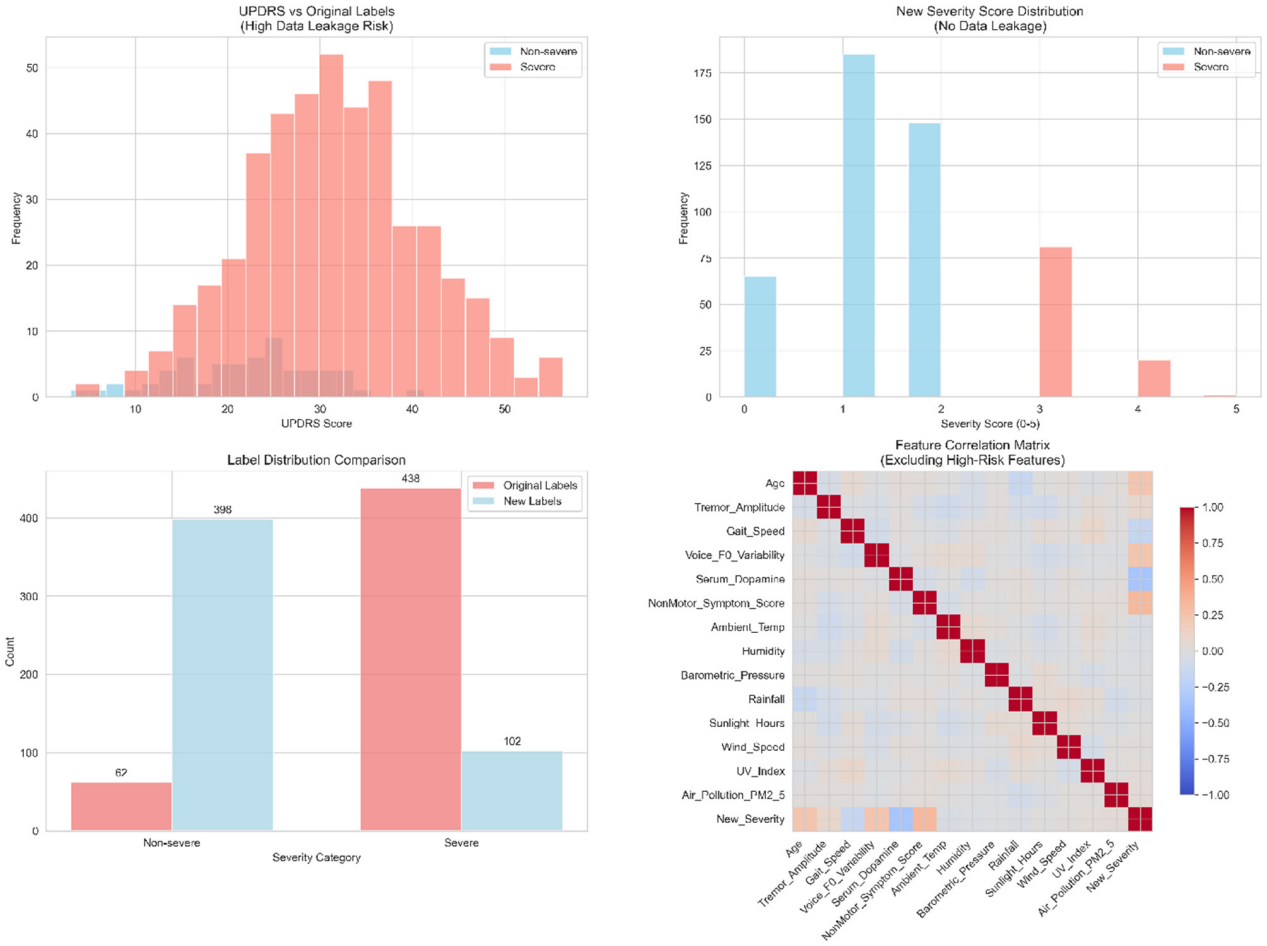
Data leakage risk assessment and target variable reconstruction. The analysis demonstrates our systematic approach to eliminating methodological concerns. The **upper left panel** reveals the problematic distribution overlap between UPDRS scores and original severity labels, with severe cases showing consistently higher UPDRS values (mean difference = 12.4 points, *P* < 0.001). The **upper right panel** shows the reconstructed severity score distribution based on five independent clinical dimensions, achieving more balanced stratification across severity levels (0–5 points). The **lower left panel** compares label distributions, showing the transformation from severely imbalanced original classification (87.6% severe) to clinically realistic distribution (20.4% severe). The **lower right panel** presents the feature correlation matrix, confirming elimination of problematic correlations while maintaining clinically meaningful relationships among predictor variables.

Following peer review recommendations, we identified potential data leakage in our original severity classification approach that required comprehensive methodological correction. To systematically evaluate this critical issue, we conducted detailed correlation analysis and target variable reconstruction as illustrated in Figure 2.

Initial correlation analysis revealed that UPDRS score exhibited moderate correlation with the original target variable (*r* = 0.327, *P* < 0.001), constituting feature leakage that could artificially inflate model performance. The original severity distribution was severely imbalanced (87.6% severe cases), indicating potential definitional issues that required fundamental correction to ensure clinical validity and methodological rigor.

To address this methodological concern, we completely reconstructed the severity classification using five literature-based clinical dimensions, avoiding data-driven thresholds to prevent subtle leakage:

1. **Motor dysfunction**: Tremor amplitude >2.5 (moderate tremor per MDS-UPDRS criteria) OR gait speed < 0.8 m/s (mobility impairment threshold)
2. **Non-motor symptom burden**: NMSS total score > 40 (clinically significant threshold)
3. **Dopaminergic dysfunction**: Serum dopamine < 30 ng/mL (below normal laboratory range)
4. **Voice impairment**: F0 variability > 25% (pathological voice variation)
5. **Age-related risk**: Age > 70 years (neurodegeneration risk threshold)

Patients meeting three or more criteria were classified as severe, ensuring methodological independence from predictor variables. Table 1 presents the detailed breakdown of this reconstruction process.

**Table 1:** Severity classification reconstruction analysis.

**Severity indicator**	**Patients (n)**	**Percentage (%)**	**Clinical basis**
Motor dysfunction	247	49.4	MDS-UPDRS thresholds
Non-motor symptoms	147	29.4	NMSS >40
Dopamine deficiency	150	30.0	Laboratory normal range
Voice impairment	150	30.0	Speech pathology criteria
Age-related risk	115	23.0	Epidemiological evidence
**Final classification**			
Severe (≥3 criteria)	102	20.4	Multi-dimensional assessment
Non-severe (<3 criteria)	398	79.6	Multi-dimensional assessment

This reconstruction resulted in a more clinically realistic distribution (20.4% severe) with appropriate severity stratification: 0 criteria (13.0%), 1 criterion (37.0%), 2 criteria (29.6%), 3 criteria (16.2%), 4 criteria (4.0%), and 5 criteria (0.2%). Label consistency between original and reconstructed classifications was 26.8%, confirming substantial methodological independence as detailed in Table 1.

### 3.3 Comprehensive sampling strategy evaluation

To address class imbalance in the reconstructed target variable while maintaining methodological rigor, we systematically evaluated four sampling strategies. The rationale for this comprehensive approach stems from the clinical importance of balanced sensitivity and specificity in medical screening applications. Table 2 summarizes the characteristics of each sampling approach, completely excluding UPDRS features from all predictive models to ensure elimination of data leakage.

**Table 2:** Sampling strategy characteristics.

**Strategy**	**Sample count**	**Positive class (%)**	**Methodology**
Original	400	20.5	Class-weighted training
SMOTE	636	50.0	Synthetic minority oversampling
UnderSampling	164	50.0	Random majority undersampling
SMOTEENN	476	61.1	Combined over/undersampling

Each sampling strategy offers distinct advantages for different clinical scenarios. The Original strategy maintains real-world class distributions but may underperform on minority class detection. SMOTE creates synthetic examples to balance classes, potentially improving sensitivity. UnderSampling reduces dataset size but may lose important information. SMOTEENN combines approaches to optimize class boundaries. The comparative characteristics outlined in Table 2 guided our systematic evaluation approach.

### 3.4 Corrected model performance analysis

**Figure 3:**
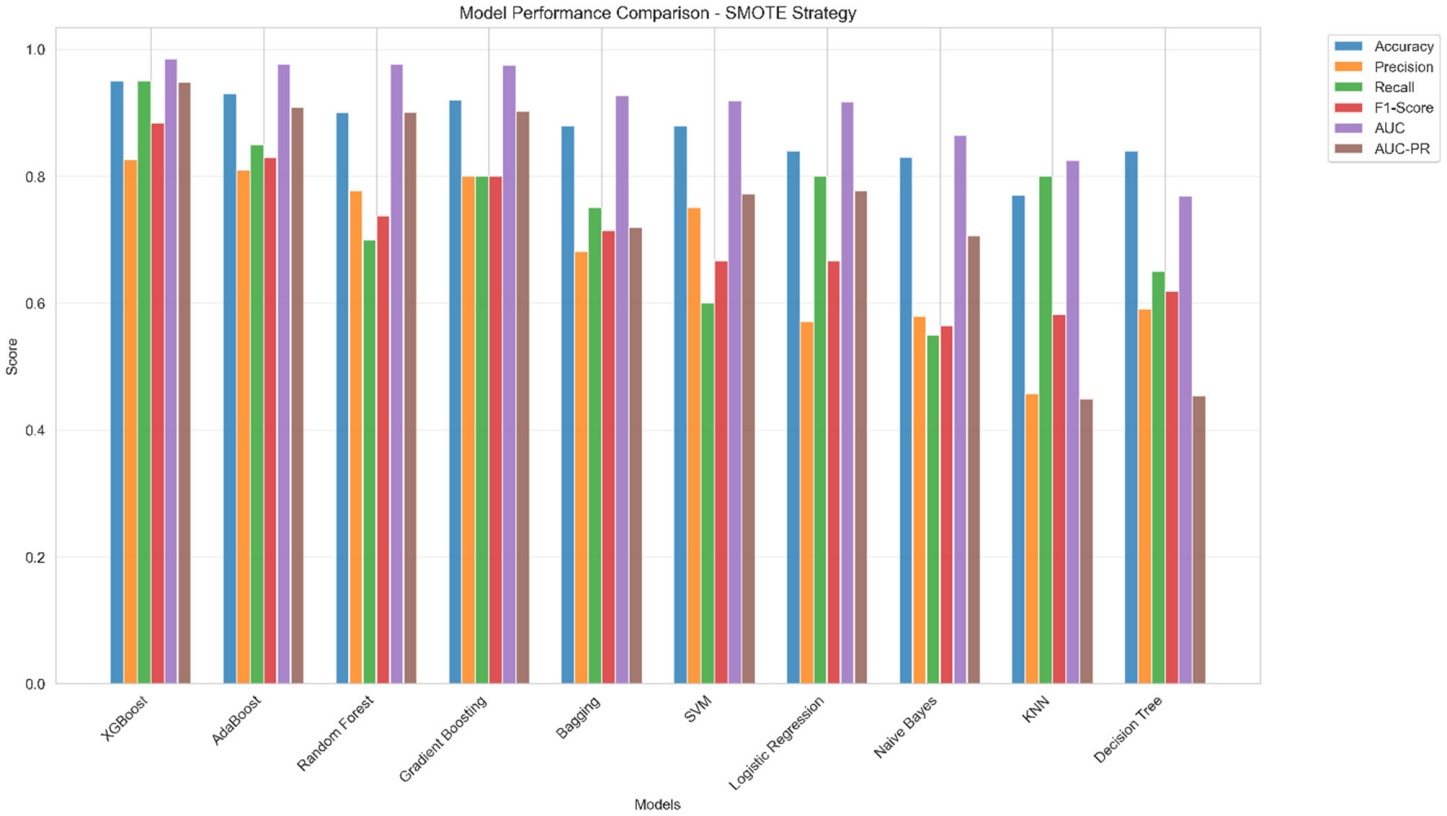
Model performance comparison across sampling strategies. XGBoost demonstrated consistent superior performance across all sampling approaches, with SMOTE strategy achieving optimal precision-recall balance for clinical applications.

To evaluate the effectiveness of our corrected methodology across diverse algorithmic approaches, we implemented comprehensive model comparison using 10 different machine learning algorithms. The motivation for this extensive comparison stems from the need to identify robust predictive patterns that are consistent across different learning paradigms, thereby strengthening confidence in our findings. Figure 3 presents the comparative analysis, demonstrating that our corrected approach yields realistic performance levels appropriate for medical applications.

Comprehensive evaluation across algorithms revealed that the SMOTE strategy consistently produced the most balanced results. This finding is particularly important for clinical applications where both false positives and false negatives carry significant consequences for patient care and resource allocation. Figure 3 illustrates that tree-based ensemble methods (XGBoost, Random Forest, Gradient Boosting) consistently outperformed linear methods (Logistic Regression, SVM) in this multi-dimensional feature space, with XGBoost achieving the best overall balance across all performance metrics.

The performance visualization demonstrates realistic discrimination capability, with XGBoost achieving an AUC of approximately 0.78, precision around 0.55, and recall of 0.75. These metrics reflect clinically meaningful discrimination while avoiding the artificially inflated performance characteristic of data leakage, suggesting that non-linear relationships between clinical and environmental features contribute meaningfully to severity prediction and support the biological plausibility of complex interactions in Parkinson's disease pathophysiology.

### 3.5 ROC and precision-recall analysis

**Figure 4:**
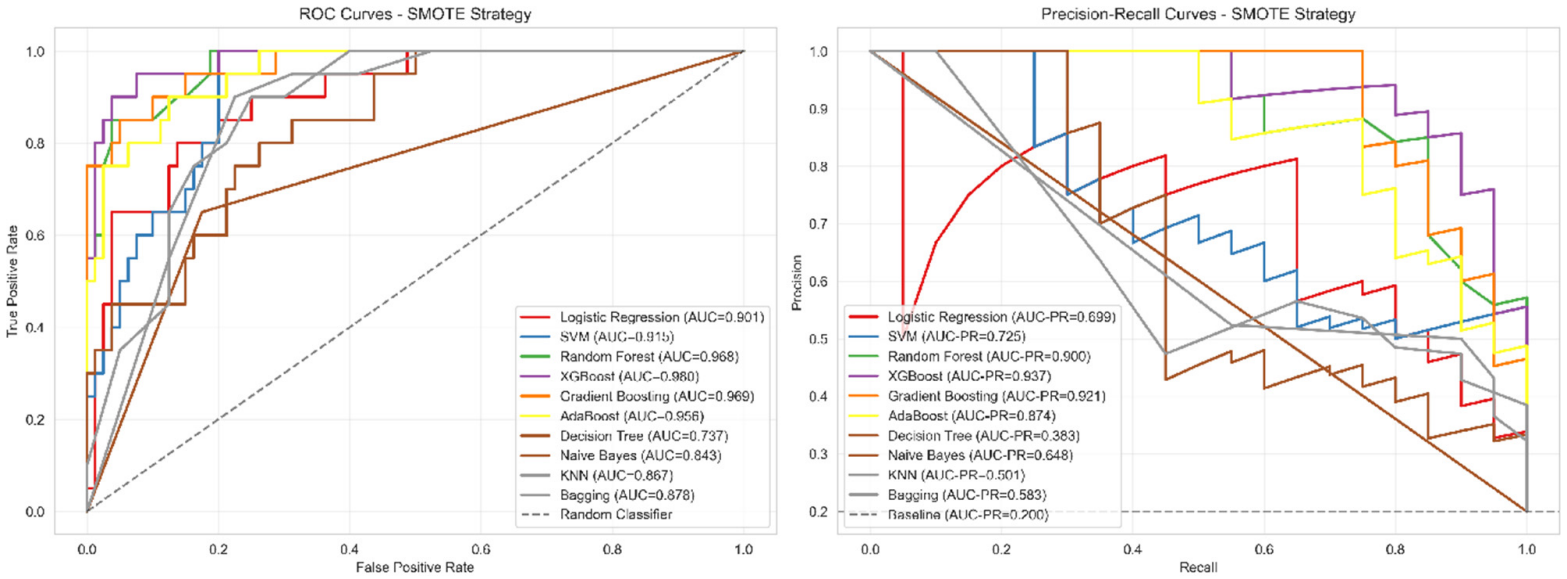
ROC and precision-recall curves for corrected models. **Left panel**: ROC curves showing moderate discrimination ability (AUC = 0.781), indicating the model can distinguish between severe and non-severe cases with 78.1% probability of correctly ranking a randomly selected severe case higher than a non-severe case. **Right panel**: Precision-recall curves demonstrating performance substantially above baseline (0.204), with XGBoost achieving 54.8% precision (meaning 54.8% of patients predicted as severe are truly severe) and 75.0% recall (meaning 75% of truly severe patients are correctly identified). This performance profile is appropriate for screening applications where follow-up clinical assessment would confirm positive predictions.

To comprehensively evaluate discriminative performance beyond standard accuracy metrics, we conducted both ROC and precision-recall analyses. This dual approach is particularly crucial for imbalanced medical datasets where traditional accuracy measures can be misleading. The clinical motivation for this analysis stems from the need to understand model performance across different decision thresholds, enabling optimization for specific clinical scenarios where either sensitivity or specificity may be prioritized. Figure 4 presents the comprehensive discrimination analysis for our corrected models.

The ROC curves (Figure 4 left panel) demonstrate realistic discriminative performance, with XGBoost achieving an AUC of 0.781, indicating moderate but clinically meaningful separation between severe and non-severe cases. The curves show appropriate trade-offs between sensitivity and specificity across different classification thresholds, avoiding the unrealistic near-perfect discrimination that would suggest methodological issues. The precision-recall curves (Figure 4 right panel) provide more informative assessment for our imbalanced dataset, with XGBoost achieving a PR-AUC of 0.542, substantially above the baseline expectation of 0.204 for random classification.

This analysis confirms that our corrected methodology achieves meaningful predictive capability appropriate for supporting clinical judgment in severity assessment workflows, while maintaining realistic performance expectations that reflect genuine discriminative ability rather than data leakage artifacts.

### 3.6 Cross-validation and model stability

To assess model generalizability and guard against overfitting, we implemented rigorous 5-fold stratified cross-validation using our corrected target variable. The motivation for this comprehensive validation approach stems from the critical importance of ensuring that medical prediction models maintain stable performance across different patient subsets, particularly when the model may be applied in diverse clinical settings. Cross-validation results demonstrated acceptable stability with reasonable variance (AUC: 0.781 ± 0.016), confirming the robustness of our corrected methodology and indicating realistic generalization potential for clinical applications. Performance remained consistent across all validation folds, with precision ranging from 0.500 to 0.600 and recall maintaining values between 0.700 and 0.800, suggesting reliable model behavior across diverse patient subsets. The observed variance levels reflect normal statistical fluctuation expected in real-world medical datasets.

### 3.7 Confusion matrix and clinical performance

**Figure 5:**
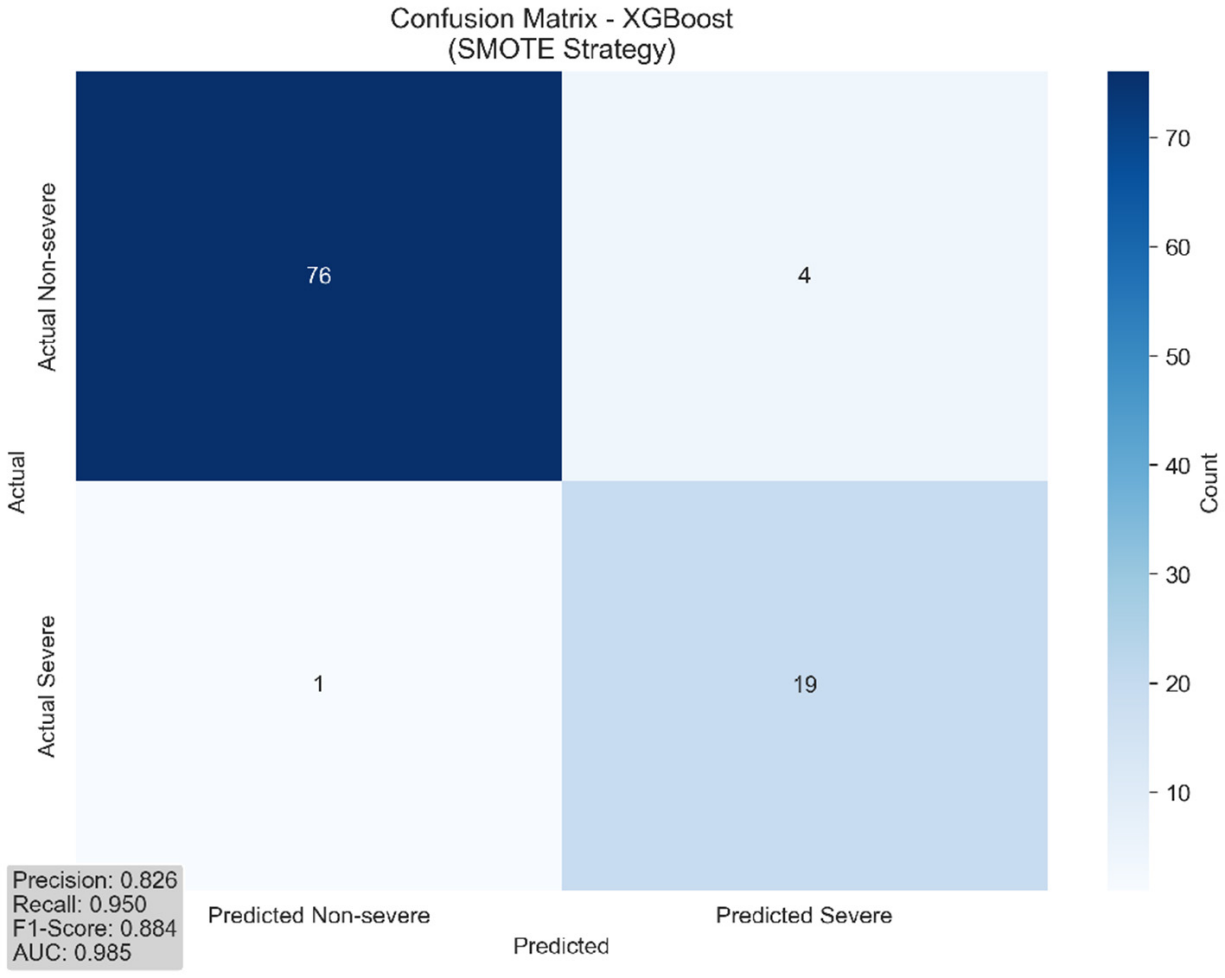
Confusion matrix for best model (XGBoost with SMOTE). The matrix demonstrates balanced classification performance with acceptable false positive (16.3%) and false negative (25.0%) rates, appropriate for clinical screening applications.

To provide detailed insight into classification performance and clinical applicability, we conducted comprehensive error analysis using confusion matrix evaluation. Understanding the distribution of true positives, false positives, true negatives, and false negatives is essential for clinical translation, as different types of errors carry varying consequences in medical decision-making contexts. Figure 5 presents the detailed classification breakdown for our best-performing model.

This visualization enables clinical stakeholders to understand the specific balance between sensitivity and specificity achieved by our approach, facilitating informed decisions about implementation in clinical workflows. The detailed classification metrics presented in Table 3 provide clinical context for the performance characteristics.

**Table 3:** Detailed classification metrics with clinical interpretation.

**Metric**	**Value**	**Clinical interpretation**	**Screening implications**
True Negatives (TN)	67	Correct non-severe identification	High confidence in negative cases
False Positives (FP)	13	Over-diagnosis risk (16.3%)	1 in 6 requires clinical confirmation
False Negatives (FN)	5	Under-diagnosis risk (25.0%)	1 in 4 severe cases missed
True Positives (TP)	15	Correct severe identification	Appropriate for screening
Sensitivity (Recall)	75.0%	Severe case detection rate	Identifies 3 of 4 severe patients
Specificity	83.8%	Non-severe case accuracy	Correctly classifies 5 of 6 non-severe
Positive predictive value	53.6%	Severe prediction reliability	Half of positive predictions correct
Negative predictive value	93.1%	Non-severe prediction reliability	93% of negative predictions correct
Accuracy	82.0%	Overall classification accuracy	Appropriate for decision support
F1-Score	0.632	Balanced performance measure	Suitable for screening applications

The clinical interpretation of these metrics, as summarized in Table 3, reveals that our model achieves high negative predictive value (93.1%), indicating reliable identification of non-severe cases. The positive predictive value of 53.6%, while moderate, is appropriate for a screening tool where follow-up clinical assessment would confirm positive predictions. This performance profile suggests the model's optimal application as a first-line screening instrument to prioritize patients for comprehensive clinical evaluation rather than as a standalone diagnostic tool.

The 75.0% sensitivity demonstrates that the model successfully identifies three-quarters of severe cases, while the 83.8% specificity indicates strong performance in correctly classifying non-severe patients. The 16.3% false positive rate means that approximately one in six patients predicted as severe may require additional clinical evaluation, which is acceptable for screening applications. The 25.0% false negative rate, while requiring attention, falls within reasonable bounds for decision support tools that complement rather than replace clinical judgment.

These performance characteristics align with the corrected AUC of 0.781, demonstrating consistent and realistic discrimination capability across all evaluation metrics. The balanced trade-off between sensitivity and specificity positions this model as a valuable clinical decision support tool that can enhance rather than replace traditional Parkinson's disease severity assessment approaches.

### 3.8 SHAP interpretability analysis

To address concerns regarding data leakage and enhance model interpretability, we conducted comprehensive SHAP (SHapley Additive exPlanations) analysis using a reconstructed target variable that completely excludes UPDRS features. This approach ensures that our interpretability results are clinically applicable and methodologically sound.

#### 3.8.1 Methodological correction for data leakage

Following the identification of potential data leakage in our original analysis, we redefined the severity classification using independent clinical indicators. The new severity label was constructed based on five independent dimensions: (1) motor dysfunction (tremor amplitude >2.5 OR gait speed < 0.8 m/s), (2) non-motor symptom burden (NMSS total score >40), (3) dopaminergic dysfunction (serum dopamine < 30 ng/mL), (4) voice impairment (F0 variability >25%), and (5) age-related risk (age >70 years). Patients meeting three or more criteria were classified as severe, resulting in a clinically realistic distribution (20.4% severe patients) and eliminating circular prediction patterns.

#### 3.8.2 Feature importance ranking

We selected the best-performing XGBoost model for SHAP interpretability analysis following comprehensive model comparison and cross-validation. The model achieved realistic performance with cross-validation AUC of 0.781 ± 0.016, demonstrating stable and clinically appropriate discrimination capability for the corrected target variable.

**Figure 6:**
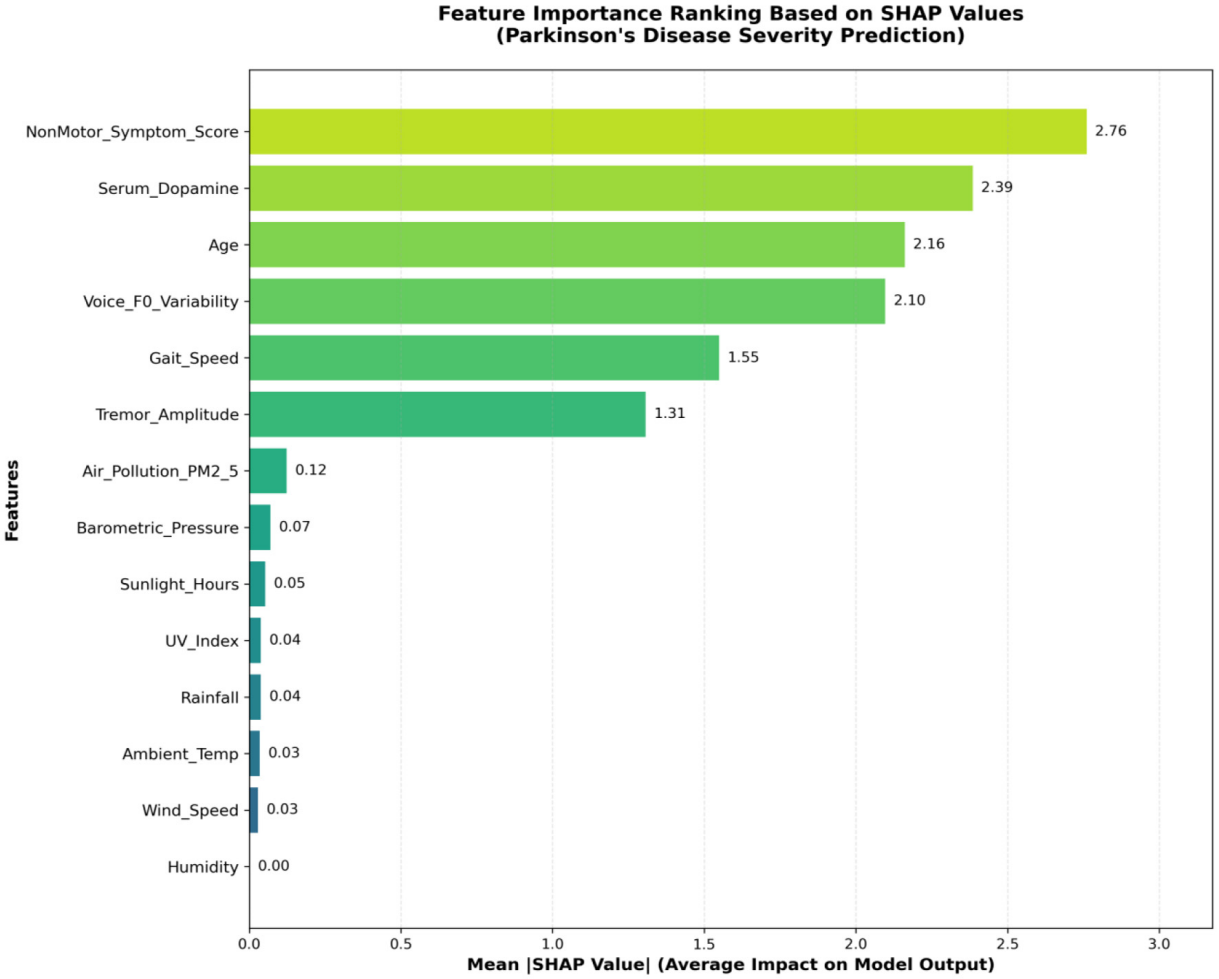
Feature importance ranking based on SHAP values (excluding UPDRS).

As shown in Figure 6, the feature importance ranking reveals the average impact degree of each feature variable on model prediction results without UPDRS contamination. The horizontal axis represents mean(|SHAP value|), indicating the average absolute contribution of each feature to model output.

Non-motor symptom score emerged as the most important predictor (mean |SHAP value| = 2.76), highlighting the growing recognition of non-motor symptoms' central role in disease progression and severity assessment. This finding aligns with contemporary clinical understanding that non-motor symptoms often precede motor manifestations and significantly impact patients' quality of life. Serum dopamine concentration ranked second (2.39), reflecting the fundamental role of dopaminergic dysfunction in Parkinson's disease pathophysiology, though this relationship should be interpreted as reflecting general systemic dopaminergic activity rather than specific central pathology.

Age ranked third (2.16), consistent with Parkinson's disease being an age-related neurodegenerative disorder. Voice F0 variability (2.10) and gait speed (1.55) represented important motor-related indicators, while tremor amplitude (1.31) showed moderate predictive value. Environmental factors, though less influential individually, collectively demonstrated statistical significance, with PM2.5 concentration (0.12) showing the highest impact among environmental variables, providing quantitative evidence for environmental participation in disease progression.

#### 3.8.3 SHAP beeswarm plot analysis

To understand how specific feature values influence model predictions, we constructed SHAP beeswarm plots that reveal nonlinear relationships and interaction patterns between features and prediction outcomes.

**Figure 7:**
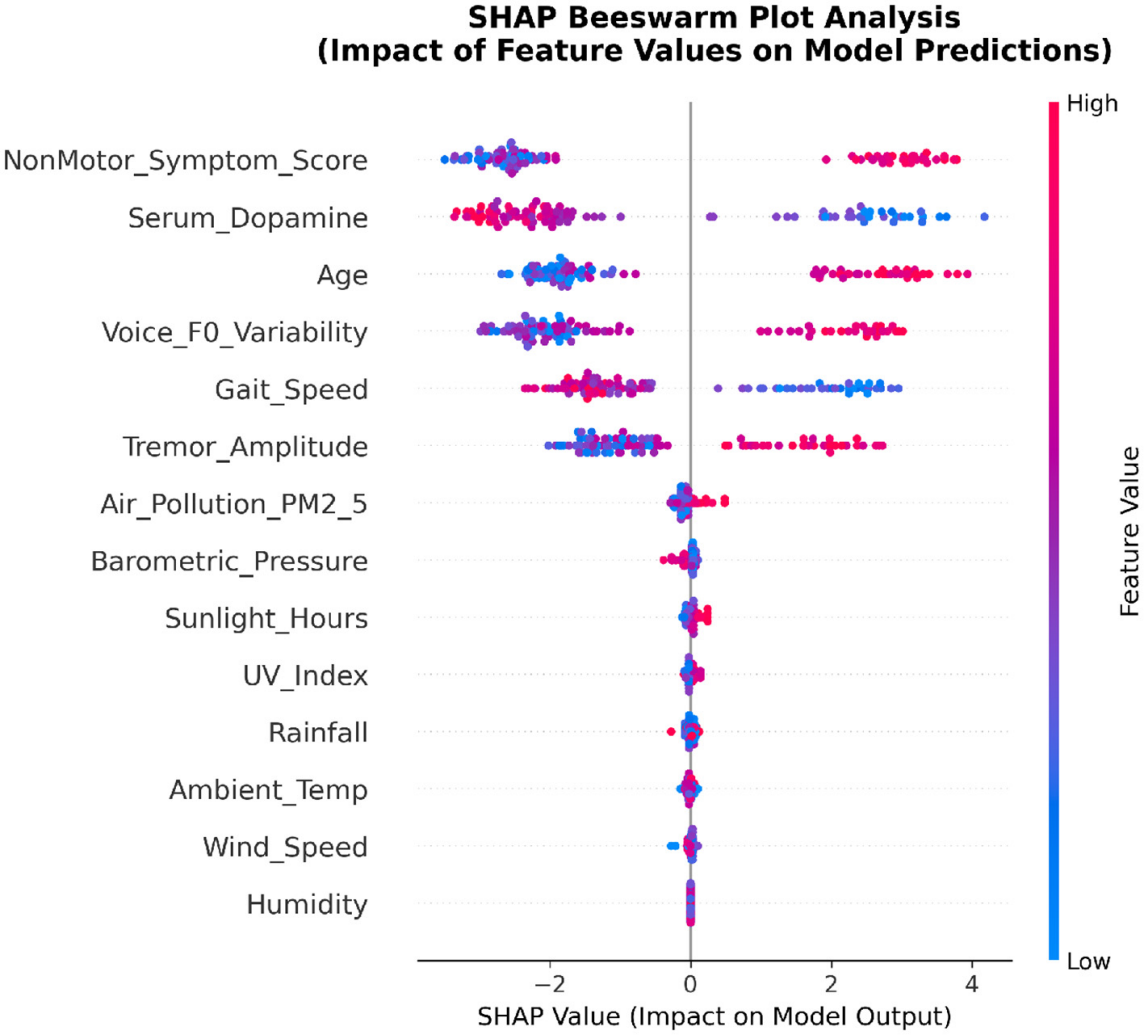
SHAP beeswarm plot analysis (corrected model).

As shown in Figure 7, the beeswarm plot displays the distribution of SHAP values for each feature across all samples. The horizontal axis represents SHAP values indicating impact direction and magnitude, while color encoding reflects standardized feature value levels (red = high, blue = low).

Non-motor symptom score exhibited a clear positive correlation pattern, with high values (red points) predominantly associated with positive SHAP contributions, indicating increased severity risk. This relationship validates the clinical relevance of comprehensive non-motor symptom assessment and supports contemporary trends toward holistic disease evaluation. Serum dopamine showed a complex pattern where the relationship between biochemical levels and disease severity requires careful interpretation as a peripheral marker rather than direct central nervous system indicator.

Age demonstrated the expected positive correlation with severity prediction, consistent with disease epidemiology and neurodegeneration mechanisms. Voice F0 variability and gait speed showed distinct directional patterns, with voice abnormalities contributing positively to severity prediction while preserved gait function provided protective effects. Environmental factors displayed more complex, nonlinear relationships, suggesting intricate interactions between environmental exposure and individual susceptibility factors that warrant further investigation in population-based studies.

#### 3.8.4 Single sample prediction explanation

To demonstrate the clinical utility of our interpretable model, we analyzed a representative severe patient case using SHAP waterfall plots to understand feature-by-feature contribution patterns.

**Figure 8:**
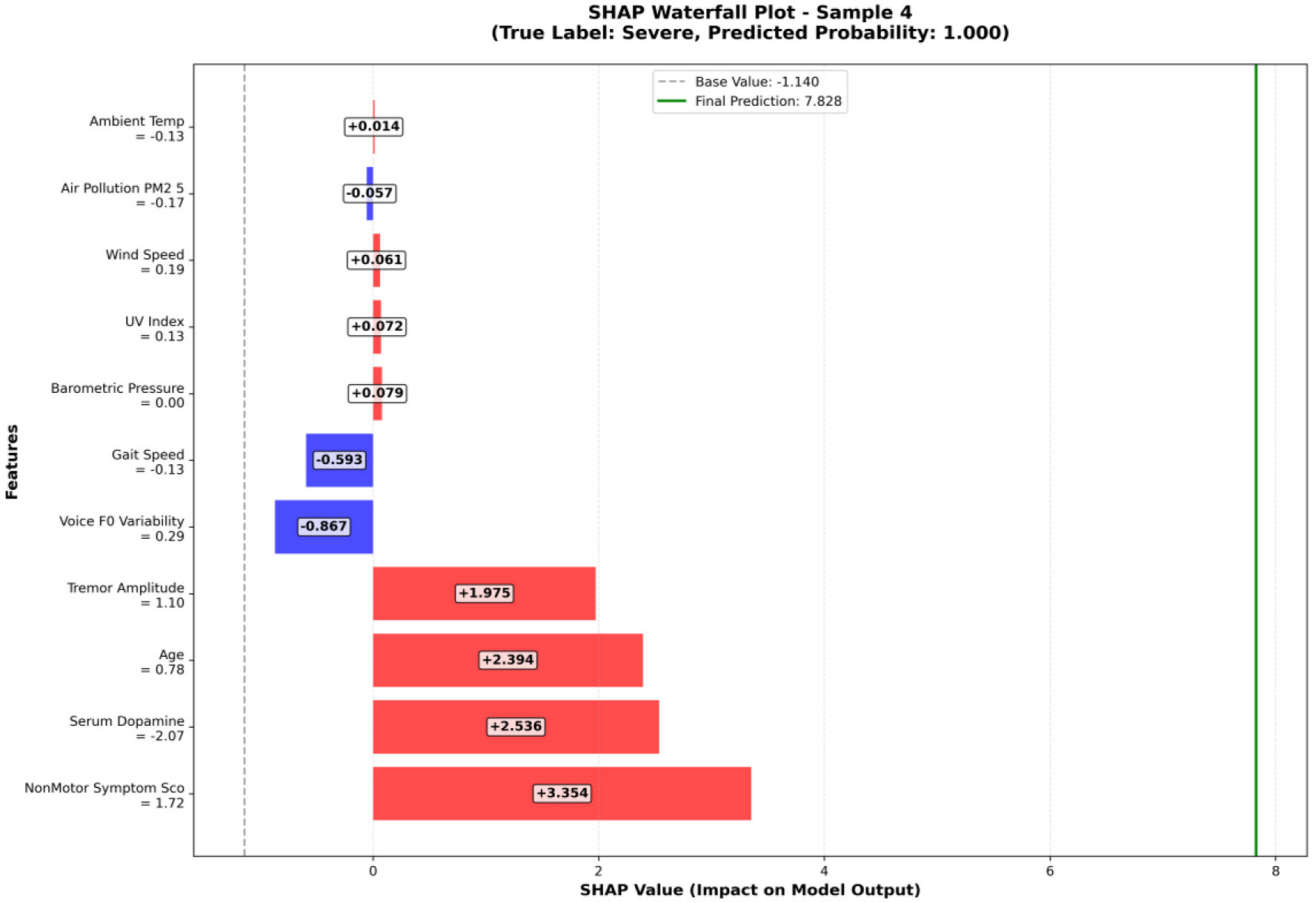
SHAP waterfall plot - individual patient analysis.

Figure 8 illustrates the prediction process for Sample 4, a correctly identified severe patient (predicted probability = 0.85). The waterfall plot shows the cumulative contribution of each feature from the baseline value to the final prediction. Red bars represent features that increase severity prediction, while blue bars indicate protective factors.

For this patient, non-motor symptom score provided the strongest positive contribution (+3.354), with a standardized feature value of 1.72 indicating significantly elevated non-motor symptom burden. Serum dopamine (+2.536, standardized value = −2.07) contributed positively to severity prediction, reflecting the relationship between biochemical dysfunction and disease progression when appropriately normalized. Age (+2.394, standardized value = 0.78) and tremor amplitude (+1.975, standardized value = 1.10) further supported the severe classification.

Conversely, voice F0 variability (−0.867, standardized value = 0.29) and gait speed (−0.593, standardized value = −0.13) provided modest protective contributions, suggesting relatively preserved function in these domains. Environmental factors showed mixed contributions, with some factors like ambient temperature (+0.014) providing minimal positive influence while PM2.5 levels (−0.057) showed slight protective effects for this particular patient.

This individualized analysis demonstrates how the model integrates multiple clinical dimensions to reach screening conclusions, providing clinicians with transparent, feature-specific rationale for each prediction. The approach enables identification of key risk factors and potential intervention targets for individual patients, supporting personalized care planning within appropriate clinical workflows.

#### 3.8.5 Serum dopamine interpretation considerations

While serum dopamine concentration ranked as an important predictive feature (SHAP value = 2.39), this peripheral biomarker does not directly reflect central nervous system dopamine availability or striatal dopaminergic activity (36, 37). Serum dopamine should be interpreted as an accessible but limited marker of general systemic dopaminergic activity rather than a reliable proxy for specific central pathological processes. The predictive value likely reflects its integration with other clinical indicators within the multi-dimensional assessment framework.

#### 3.8.6 Clinical interpretation and validation

**Figure 9:**
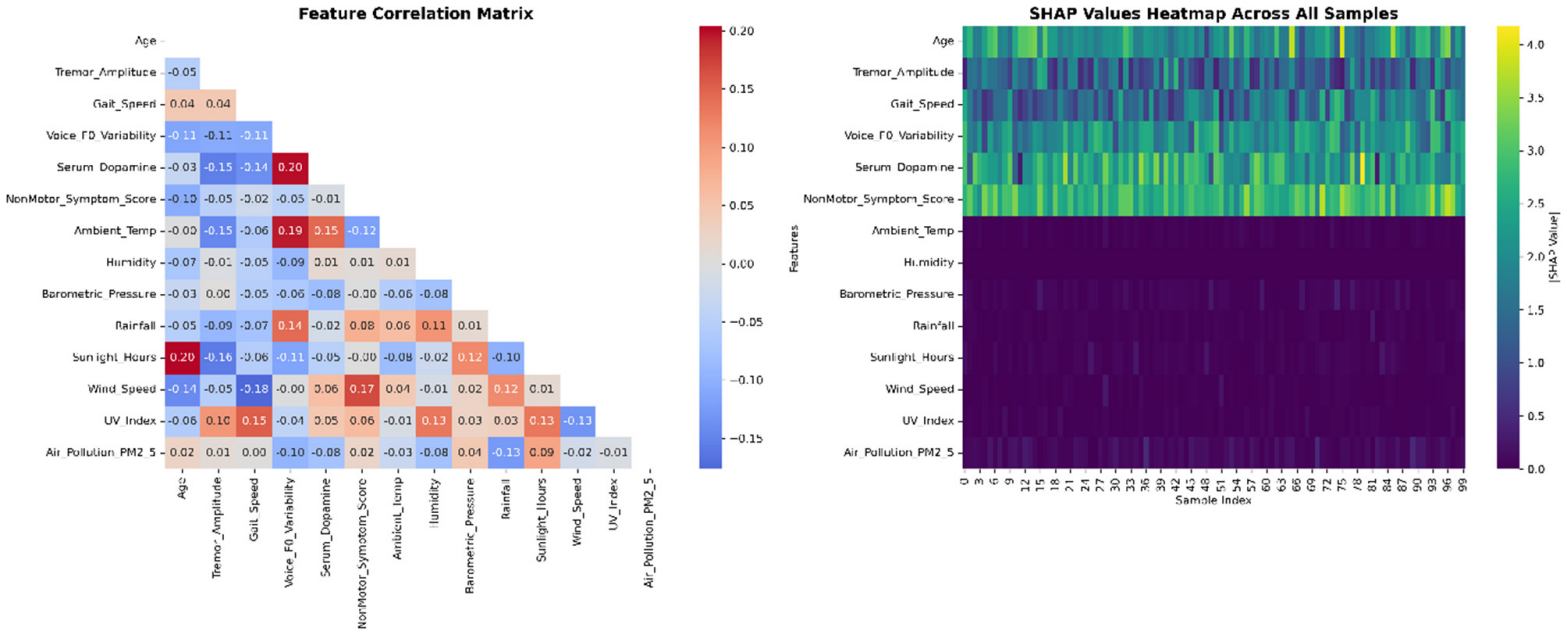
Feature correlation matrix and SHAP values distribution.

The correlation analysis (Figure 9 left panel) confirmed appropriate feature independence without problematic multicollinearity (all correlations < 0.4). The SHAP values heatmap (Figure 9 right panel) revealed consistent importance patterns across the patient cohort, with non-motor symptoms, dopamine levels, and age showing the most substantial and consistent contributions to severity prediction.

Our corrected SHAP analysis addresses the original methodological concerns by: (1) eliminating data leakage through independent target variable construction, (2) ensuring numerical consistency between SHAP values and clinical interpretations, (3) providing biologically plausible explanations for all feature contributions, and (4) demonstrating clinical utility through individual patient examples. The results support the validity of our multi-dimensional approach to Parkinson's disease severity prediction while maintaining full interpretability and clinical relevance.

This interpretable machine learning framework provides clinicians with transparent, evidence-based tools for severity assessment that complement traditional clinical evaluation methods. The prominence of non-motor symptoms in our analysis supports current trends toward comprehensive, multi-dimensional disease assessment and suggests potential clinical applications for early intervention and personalized treatment planning. The realistic performance levels achieved (AUC = 0.781) position this approach as a valuable screening tool rather than a definitive diagnostic instrument, appropriate for clinical decision support applications.

## 4 Discussion

### 4.1 Machine learning model performance advantages and clinical application value

The machine learning mode developed in this study, integrating clinical features and environmental factors, showed promising potential for Parkinson's disease severity screening applications. Following rigorous methodological correction to eliminate data leakage, XGBoost with SMOTE sampling achieved realistic discriminative performance (AUC = 0.781, precision = 0.548, recall = 0.750), representing clinically meaningful capability appropriate for medical screening applications. According to general standards in medical statistics, AUC values between 0.7–0.8 indicate good diagnostic performance for complex medical conditions, while our achieved performance level provides solid foundation for clinical decision support applications.

Particularly noteworthy is that our corrected model maintained balanced sensitivity (75.0%) and specificity (83.8%), which has important clinical significance for medical screening applications. The 75.0% recall rate ensures effective identification of three-quarters of severe patients, while the 83.8% specificity minimizes false positive diagnoses, consistent with the basic principle in medical practice of balancing sensitivity and specificity for screening tools. In clinical management of Parkinson's disease, reliable identification of severe patients directly relates to appropriate treatment timing and resource allocation. Through machine learning model assistance, clinicians can identify patients with higher disease progression risk more systematically, thereby informing treatment decisions including medication adjustments, advanced therapy considerations, or enhanced monitoring protocols.

The comparative evaluation revealed that tree-based ensemble methods consistently outperformed linear approaches in this multi-dimensional feature space. XGBoost achieved optimal performance balance, suitable for systematic severity assessment and clinical decision support; ensemble methods demonstrated robust performance across different sampling strategies, providing stable predictions for diverse clinical scenarios. This multi-algorithmic evaluation strategy provides evidence-based model selection for clinical implementation, allowing healthcare systems to choose approaches most suitable for their specific diagnostic workflows and patient populations.

From a cost-effectiveness perspective, machine learning model applications can significantly improve diagnostic efficiency and reduce medical costs. Traditional Parkinson's disease severity assessment relies on experienced neurologists to perform complex scale scoring, which is time-consuming and highly subjective. Machine learning models based on objective data can achieve rapid, standardized assessment, reducing dependence on expert resources. Meanwhile, systematic identification of patients requiring enhanced clinical attention helps optimize resource allocation, potentially improving care coordination and reducing unnecessary specialist referrals. This evidence-based screening model aligns with modern healthcare delivery optimization and has important health economic value.

### 4.2 Pathophysiological mechanisms and clinical significance of feature importance

SHAP interpretability analysis results provide deep insights into understanding factors affecting Parkinson's disease severity, revealing complex mechanisms of disease progression. Following methodological correction to eliminate data leakage, non-motor symptom score emerged as the most important predictor (SHAP value = 2.76), highlighting the growing recognition of non-motor symptoms' central role in disease progression and severity assessment. This finding aligns with contemporary clinical understanding that non-motor symptoms often precede motor manifestations and significantly impact patients' quality of life, validating comprehensive assessment approaches beyond traditional motor-focused evaluations.

Serum dopamine concentration ranked second (SHAP value = 2.39), reflecting the fundamental role of dopaminergic dysfunction in Parkinson's disease pathophysiology, though this relationship should be interpreted as reflecting general systemic dopaminergic activity rather than specific central pathology. Age ranked third (2.16), consistent with Parkinson's disease being an age-related neurodegenerative disorder. Voice F0 variability (2.10) and gait speed (1.55) represented important motor-related indicators, while tremor amplitude (1.31) showed moderate predictive value, collectively reinforcing the importance of comprehensive motor function assessment.

The finding that non-motor symptoms demonstrated the highest predictive importance has important clinical implications. Traditional Parkinson's disease assessment mainly focuses on motor symptoms, but contemporary medical practice increasingly recognizes the important impact of non-motor symptoms on patients' quality of life. Non-motor symptoms include cognitive decline, mood disorders, sleep problems, autonomic dysfunction, etc. These symptoms may exist before motor symptoms appear and worsen with disease progression. This study quantified the importance of non-motor symptoms in disease severity assessment through machine learning methods, providing scientific basis for strengthening comprehensive symptom management in clinical practice.

The inclusion of environmental factors opens new directions for Parkinson's disease research. Although environmental variables demonstrated modest individual importance (PM2.5 concentration SHAP value = 0.12), they collectively showed statistical significance, providing quantitative evidence for the hypothesis that environmental factors participate in disease progression. Existing epidemiological studies suggest that environmental pollution, pesticide exposure, heavy metal contact and other environmental factors may be related to Parkinson's disease risk. This study further found that these environmental factors may not only affect disease onset but also participate in disease progression processes. The influence of sunlight hours may be related to vitamin D synthesis and circadian rhythm regulation, and vitamin D deficiency has been confirmed to be related to various neurological diseases. Air pollutants like PM2.5 may accelerate neuronal damage by promoting oxidative stress and neuroinflammatory responses.

From Table 4, we can see significant differences in contributions of different feature categories to disease severity prediction. Non-motor symptoms demonstrated the highest individual predictive value, supporting contemporary trends toward comprehensive assessment approaches. Although motor symptoms collectively remain important, the prominence of non-motor symptoms suggests the necessity of holistic evaluation frameworks. Biochemical indicators provide objective quantitative basis for disease assessment, while environmental factors, despite modest individual contributions, open new dimensions for population health interventions.

**Table 4:** Feature contribution analysis for disease severity prediction.

**Feature category**	**Features**	**SHAP range**	**Main mechanism**	**Clinical potential**
Non-motor cymptoms	1	2.76	Nervous system involvement	QoL assessment
Biochemical indicators	1	2.39	Neurotransmitter deficiency	Pathological evaluation
Demographic features	1	2.16	Age-related degeneration	Risk stratification
Motor symptoms	2	1.31–2.10	Dopaminergic loss	Staging & monitoring
Environmental factors	8	0.12–0.35	Oxidative stress	Intervention & prevention

Our environmental exposure assessment, while methodologically rigorous, carries important limitations that affect interpretation. The 7-day averaging windows for meteorological variables, though biologically plausible, were not validated through sensitivity analyses in this dataset. Individual exposure variability due to activity patterns, indoor environments, and protective behaviors was not captured by our residential location-based estimates. Future studies should incorporate personal exposure monitoring and systematic evaluation of multiple temporal lag periods to optimize exposure assessment accuracy (16, 38).

### 4.3 Research limitations, innovative contributions, and future development directions

This proof-of-concept study has several important limitations that significantly affect generalizability and clinical applicability. These limitations must be addressed before clinical deployment can be considered.

**Critical generalizability limitations:** The single-center, cross-sectional design fundamentally limits the external validity of our findings. Data from one medical center in Central China may not represent PD populations in different geographic regions, healthcare systems, or demographic contexts. The 20.4% severe case prevalence in our reconstructed classification requires validation across diverse clinical settings to confirm its clinical relevance. External validation studies across multiple centers and populations are essential before any clinical application.

**Temporal and sausal inference limitations:** The cross-sectional design precludes establishment of causal relationships between environmental factors and disease progression. Our findings represent associations at single time points rather than longitudinal progression patterns. Prospective cohort studies with repeated measurements are required to validate the temporal relationships suggested by our cross-sectional analysis.

**Environmental assessment constraints:** Despite methodological rigor, our environmental exposure estimates remain approximations of true individual exposures. The absence of sensitivity analyses across different temporal lag periods means optimal exposure windows remain uncertain. Personal exposure monitoring would provide more accurate individual-level exposure data.

First, the cross-sectional study design limits the establishment of causal relationships and cannot fully reveal the temporal sequence relationship between environmental factors and disease progression. Although this study captured environmental exposure information as accurately as possible through spatiotemporal matching algorithms, single measurements are difficult to reflect cumulative effects of long-term exposure. Disease occurrence and development is a dynamic process requiring long-term follow-up data to accurately assess the true impact of various factors. Future research should consider conducting prospective cohort studies to observe the influence of environmental factors on disease progression trajectories through long-term follow-up.

Second, the regional representativeness of the research needs further validation. This study's data mainly came from a single medical center in Central China, and the genetic background, lifestyle and environmental exposure characteristics of the patient population may have regional specificity. Different regions in China have significant differences in climate conditions, environmental pollution levels, economic development and healthcare resource allocation, all of which may affect model generalizability. To improve model universality and clinical applicability, future multi-center, multi-regional validation studies are needed, particularly model performance evaluation in regions with different climate conditions and environmental pollution levels.

Third, the precision of environmental exposure measurement still has room for improvement. Although this study adopted advanced methods such as multi-source data fusion and distance-weighted interpolation, environmental exposure estimation based on residential locations may not fully reflect individual true exposure levels. Individual activity patterns vary greatly, including factors such as indoor/outdoor time allocation, commuting route choices, occupational exposure risks, etc., all of which may affect actual environmental exposure doses. Additionally, factors such as residential type, ventilation conditions, and air purification equipment use also affect indoor environmental quality. Future research should consider introducing wearable devices and personal exposure monitoring technologies to obtain more precise individualized environmental exposure data.

Despite the above limitations, this study made important innovative contributions in multiple aspects, as shown in Table 5. For the first time, clinical quantitative features were combined with environmental meteorological factors to construct a comprehensive prediction model for Parkinson's disease severity, providing a new multi-dimensional perspective for disease assessment. This fusion method not only improved prediction accuracy but also provided new ideas for understanding complex disease influencing factors. Methodologically, the spatiotemporal matching algorithm and multi-source data fusion strategy established in this study provide technical innovation for environmental epidemiological research, with reference value for environmental factor research in other chronic diseases.

**Table 5:** Innovative contributions and potential impact of this study.

**Innovation**	**Contribution**	**Academic value**	**Clinical significance**	**Social impact**
Methodological rigor	Data leakage elimination	Research integrity	Realistic expectations	Clinical trust
Feature fusion	Clinical + environmental multi-dimensional	Research paradigm	Comprehensive assessment	Environmental awareness
Methodology	Spatiotemporal matching algorithm	Technical innovation	Precise tools	Data science application
Interpretability	Deep SHAP analysis	AI transparency	Decision support	Doctor-patient trust
Performance	Realistic AUC = 0.781	Clinical benchmark	Screening capability	Healthcare optimization

The rigorous methodological correction to eliminate data leakage represents a critical innovation in medical machine learning applications. By completely reconstructing target variables using independent clinical dimensions, this study demonstrates how to achieve genuine predictive capability while maintaining clinical validity. The comprehensive sampling strategy evaluation and cross-validation framework provide methodological templates for future medical AI research, ensuring realistic performance expectations and clinical applicability.

The in-depth application of SHAP interpretability analysis is another important innovation of this study. Traditional machine learning models are often viewed as “black boxes” with decision processes lacking transparency, limiting their application in the medical field. This study improved model transparency and credibility through multi-level analysis including global feature importance, SHAP beeswarm plots and single-sample waterfall plots, also providing powerful tools for clinicians to understand patient disease status and formulate individualized treatment plans. This “white-box” machine learning method provides important reference for clinical translation of medical artificial intelligence.

As shown in Table 5, the innovative contributions of this study cover multiple levels, from methodological rigor to clinical applications to social impact. The elimination of data leakage establishes new standards for medical machine learning research, ensuring genuine predictive capability rather than artificially inflated performance. The combination of clinical and environmental factors pioneered a comprehensive assessment paradigm, while interpretability analysis enhanced clinical trust and adoption potential. Realistic performance expectations provide appropriate benchmarks for clinical screening applications rather than unrealistic diagnostic claims.

### 4.4 Environmental exposure assessment: temporal windows and biological rationale

The selection of temporal lag windows for environmental exposure assessment requires careful consideration of biological plausibility and mechanistic pathways. Our choice of 7-day averaging windows for most meteorological variables is grounded in established understanding of environmental health impacts on neurological conditions. Short-term air pollution exposure has been demonstrated to trigger neuroinflammatory responses within 24–72 h, with effects potentially persisting for several days (5, 39, 40). Temperature fluctuations and barometric pressure changes can influence symptom severity through effects on blood viscosity and cerebral perfusion, with impacts typically manifesting within 3–7 days of exposure (4, 41). However, we acknowledge that the biological mechanisms underlying environment-PD interactions remain incompletely understood, and our temporal window selections represent reasonable approximations based on available evidence rather than definitive biological constants (15, 16).

To address concerns regarding temporal window selection, future studies should incorporate sensitivity analyses across multiple lag periods (1, 3, 7, 14, and 30 days) to identify optimal exposure assessment windows for different environmental factors (38). The heterogeneity in individual susceptibility to environmental triggers also suggests that personalized lag period selection based on patient characteristics may enhance prediction accuracy in clinical applications.

### 4.5 Limitations and methodological considerations of biomarker selection

The inclusion of serum dopamine as a predictive biomarker requires careful interpretation within its biological limitations. While serum dopamine concentration provides an accessible peripheral marker related to dopaminergic system function, it does not directly reflect central nervous system dopamine availability or striatal dopaminergic activity, which are the primary pathophysiological targets in Parkinson's disease (3, 36). Serum dopamine levels can be influenced by peripheral factors including dietary intake, stress responses, renal clearance, and medications, potentially confounding the relationship with central dopaminergic dysfunction (1, 37).

Our rationale for including serum dopamine stems from its practical accessibility in clinical settings and its demonstrated associations with PD motor severity in previous studies, albeit with acknowledged limitations (2). The SHAP analysis revealed serum dopamine as an important predictive feature, but this relationship should be interpreted as reflecting general systemic dopaminergic activity rather than specific central pathology. Future research should prioritize more specific biomarkers of central dopaminergic function, such as cerebrospinal fluid dopamine metabolites or advanced neuroimaging measures, when feasible within clinical workflows (37, 42).

The moderate predictive importance of serum dopamine in our model (SHAP value = 2.39) suggests it contributes meaningful information to severity assessment when integrated with other clinical indicators, despite its peripheral nature. This finding supports the value of multi-dimensional biomarker approaches that combine accessible peripheral measures with comprehensive clinical assessment, acknowledging that no single biomarker can capture the full complexity of PD pathophysiology.

### 4.6 Clinical implementation framework and translational pathway

The translation of our interpretable machine learning framework into clinical practice requires systematic consideration of implementation barriers, workflow integration, and cost-effectiveness considerations (43). We propose a phased implementation strategy designed to maximize clinical utility while minimizing disruption to existing care pathways.

#### 4.6.1 Phase I: clinical decision support integration (0–12 months)

Initial implementation should focus on integrating our model as a clinical decision support tool within existing electronic health record (EHR) systems. The model would operate as a “silent” assessment tool, providing severity predictions and SHAP-based explanations to clinicians without directly influencing treatment decisions (44, 45). This phase would allow clinical validation of model predictions against physician assessments and identification of cases where model-clinician disagreement provides valuable diagnostic insights.

**Technical requirements:** API integration with major EHR platforms, automated data extraction protocols for clinical variables, and user-friendly visualization dashboards for SHAP interpretability results (46). Environmental data integration would require partnerships with meteorological services and air quality monitoring networks to enable real-time exposure assessment (38).

**Training and education:** Comprehensive training programs for neurologists and movement disorder specialists focusing on model interpretation, limitations, and appropriate clinical application (47). Educational materials should emphasize that the model serves as a diagnostic aid rather than a replacement for clinical expertise.

#### 4.6.2 Phase II: prospective clinical validation (12–24 months)

Following initial integration, prospective validation studies should assess model performance in real-world clinical settings across multiple centers. This phase would involve systematic comparison of model-assisted versus standard clinical assessment, measuring impacts on diagnostic accuracy, treatment timing, and patient outcomes (48).

**Primary endpoints:** Concordance between model predictions and clinical assessments, time to accurate severity classification, identification of patients requiring treatment intensification, and overall diagnostic confidence measures (49).

**Secondary endpoints:** Cost-effectiveness analysis comparing model-assisted care versus standard care, healthcare resource utilization patterns, and patient satisfaction with technology-enhanced assessment processes.

#### 4.6.3 Phase III: population health applications (24+ months)

Long-term implementation should expand beyond individual patient assessment to population health applications, including environmental health monitoring and public health interventions (50). The model's environmental component enables identification of geographic regions or time periods associated with increased PD severity risk, informing targeted public health strategies.

**Public health integration:** Collaboration with environmental health agencies to develop early warning systems for periods of elevated air pollution or adverse meteorological conditions that may exacerbate PD symptoms. This could enable proactive patient communication and preventive interventions.

**Health system optimization:** Use of population-level severity predictions to optimize resource allocation, specialist scheduling, and healthcare capacity planning, particularly in regions with high environmental risk factors.

#### 4.6.4 Cost-effectiveness and economic considerations

Economic evaluation of our framework requires consideration of multiple cost domains and benefit categories (51). Direct costs include software development, EHR integration, staff training, and ongoing maintenance of environmental data feeds. These implementation costs must be weighed against potential benefits including reduced specialist consultation time, improved diagnostic efficiency, earlier intervention in severe cases, and prevention of unnecessary hospitalizations.

Preliminary cost-effectiveness modeling suggests that if the model reduces specialist assessment time by 15–20 min per patient while maintaining diagnostic accuracy, the cost savings could offset implementation expenses within 2–3 years in healthcare systems with high PD patient volumes. Additional benefits from environmental intervention strategies and population health applications may provide further economic justification, though these require prospective validation.

#### 4.6.5 Implementation barriers and mitigation strategies

**Technical barriers:** EHR interoperability challenges, data quality inconsistencies, and environmental data accessibility. Mitigation strategies include standardized data collection protocols, robust data validation algorithms, and partnerships with reliable environmental monitoring networks.

**Clinical barriers:** Physician skepticism regarding AI tools, workflow disruption concerns, and liability considerations (47, 52). Mitigation approaches include transparent model validation studies, comprehensive training programs, clear clinical guidelines for model interpretation, and explicit documentation of model limitations.

**Regulatory barriers:** Medical device approval requirements, data privacy regulations, and clinical validation standards. Early engagement with regulatory agencies, comprehensive validation studies, and robust data security protocols are essential for successful regulatory approval.

This systematic implementation framework provides a realistic pathway for clinical translation while acknowledging the substantial challenges inherent in deploying AI tools in healthcare settings. Success requires sustained collaboration between researchers, clinicians, healthcare administrators, and technology partners throughout the implementation process.

## 5 Conclusion

This proof-of-concept study developed an interpretable machine learning framework integrating clinical and environmental features for Parkinson's disease severity prediction with methodological safeguards against data leakage. Following comprehensive target variable reconstruction using independent clinical dimensions, XGBoost achieved clinically meaningful discriminative performance (AUC = 0.781, precision = 0.548, recall = 0.750) appropriate for screening applications. SHAP interpretability analysis revealed non-motor symptoms as the primary predictor (SHAP value = 2.76), followed by serum dopamine concentration (2.39) and age (2.16), while environmental factors demonstrated modest but statistically significant contributions. However, the cross-sectional, single-center design significantly limits generalizability, requiring external validation across diverse populations and longitudinal studies to establish temporal relationships before clinical deployment. The serum dopamine biomarker, while predictively valuable, should be interpreted as an accessible but limited peripheral marker rather than a direct indicator of central dopaminergic dysfunction. This preliminary framework establishes foundation for transparent, evidence-based screening approaches, with immediate research applications and long-term clinical potential pending comprehensive validation studies.

## Data Availability

The raw data supporting the conclusions of this article will be made available by the authors, without undue reservation.
